# Establishing Cancer Treatment Programs in Resource-Limited Settings: Lessons Learned From Guatemala, Rwanda, and Vietnam

**DOI:** 10.1200/JGO.17.00082

**Published:** 2018-08-07

**Authors:** Claire M. Wagner, Federico Antillón, François Uwinkindi, Tran Van Thuan, Sandra Luna-Fineman, Pham Tuan Anh, Tran Thanh Huong, Patricia Valverde, Arielle Eagan, Pham Van Binh, Tien Nguyen Quang, Sonali Johnson, Agnes Binagwaho, Julie Torode

**Affiliations:** **Claire M. Wagner**, **Arielle Eagan**, and **Agnes Binagwaho**, Harvard Medical School, Boston, MA; **Sonali Johnson** and **Julie Torode**, Union for International Cancer Control, Geneva, Switzerland; **Federico Antillón,** Unidad Nacional de Oncología Pediatrica; and Universidad Francisco Marroquín; **Patricia Valverde**, Unidad Nacional de Oncología Pediatrica, Guatemala City, Guatemala; **François Uwinkindi** and and **Arielle Eagan**, Rwanda Biomedical Center; **Agnes Binagwaho**, University of Global Health Equity, Kigali, Rwanda; **Tran Van Thuan**, **Pham Tuan Anh**, **Tran Thanh Huong**, **Pham Van Binh**, and **Nguyen Tien Quang**, National Cancer Hospital of Viet Nam; **Tran Van Thuan** and **Tran Thanh Huong**, National Institute for Cancer Control; **Tran Thanh Huong**, Hanoi Medical University, Hanoi, Viet Nam; **Sandra Luna-Fineman**, Children's Hospital Colorado and University of Colorado, Denver, CO; and **Agnes Binagwaho and Arielle Eagan**, Dartmouth College, Hanover, NH.

## Abstract

**Purpose:**

The global burden of cancer is slated to reach 21.4 million new cases in 2030 alone, and the majority of those cases occur in under-resourced settings. Formidable changes to health care delivery systems must occur to meet this demand. Although significant policy advances have been made and documented at the international level, less is known about the efforts to create national systems to combat cancer in such settings.

**Methods:**

With case reports and data from authors who are clinicians and policymakers in three financially constrained countries in different regions of the world—Guatemala, Rwanda, and Vietnam, we examined cancer care programs to identify principles that lead to robust care delivery platforms as well as challenges faced in each setting.

**Results:**

The findings demonstrate that successful programs derive from equitably constructed and durable interventions focused on advancement of local clinical capacity and the prioritization of geographic and financial accessibility. In addition, a committed local response to the increasing cancer burden facilitates engagement of partners who become vital catalysts for launching treatment cascades. Also, clinical education in each setting was buttressed by international expertise, which aided both professional development and retention of staff.

**Conclusion:**

All three countries demonstrate that excellent cancer care can and should be provided to all, including those who are impoverished or marginalized, without acceptance of a double standard. In this article, we call on governments and program leaders to report on successes and challenges in their own settings to allow for informed progression toward the 2025 global policy goals.

## INTRODUCTION

The global burden of cancer is expected to increase significantly by 2030; in that year alone, an estimated 21.4 million new cases and 13.2 million deaths will occur, and the vast majority of this burden will befall low- and middle-income countries.^1-4^ In all regions of the world, formidable changes to health care delivery systems must occur to meet this demand. Although global goals and targets have been agreed upon at the international level,^5-11^ less has been documented about the principles that underlie national efforts to grow cancer treatment programs. As such, implementation blueprints for a quality cancer system remain scarce, as clinicians and policy-makers are strapped by incommensurate human resource capacity, infrastructure, treatment options, financial support, and—often—political will.^12^

However, promising examples of comprehensive treatment programs are emerging within under-resourced health systems. Our aims in this article are to describe three distinct care delivery models in Guatemala, Rwanda, and Vietnam and to illuminate the strategies that allowed each program to thrive in its own context. Each country case begins with a concise account of the national background, which is followed by an illustration of cancer planning and health system integration. Table 1 and Table 2 provide demographic and cancer burden profiles, respectively. The Panel shown in this article uses the WHO’s “Six Building Blocks for Health Systems”^13^ to synthesize shared elements among the three cancer programs. The Discussion distills lessons learned: principles and themes that have led to success as well as roadblocks and remaining challenges.

**Table 1:**
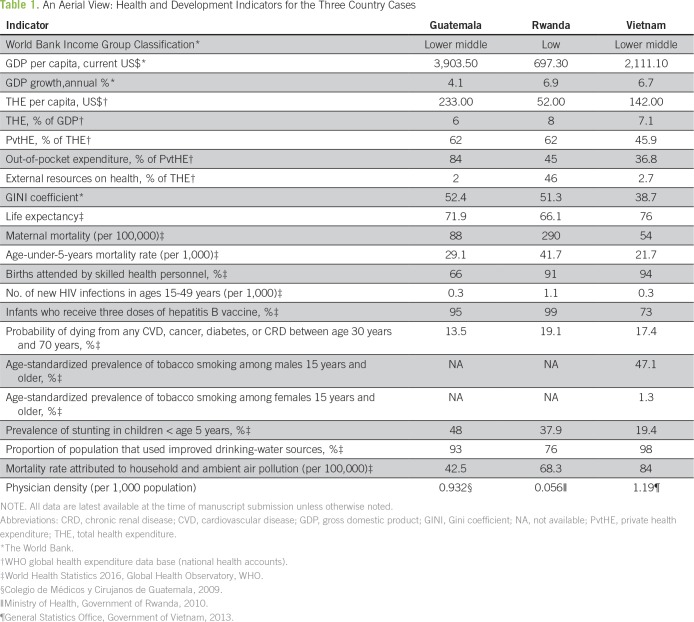
An Aerial View: Health and Development Indicators for the Three Country Cases

**Table 2:**
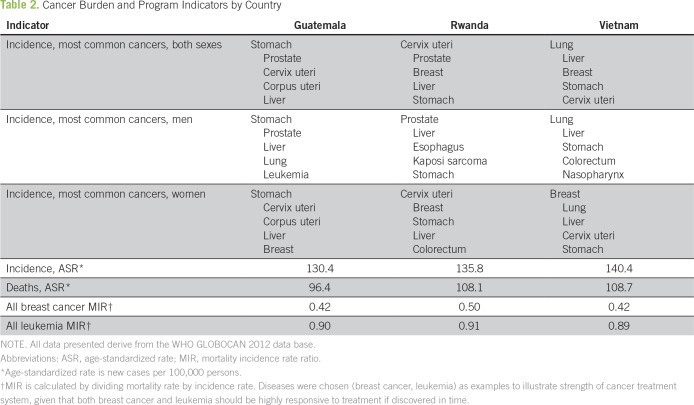
Cancer Burden and Program Indicators by Country

These cases are singular and not meant to be representative. Rather, they allow us to extract translatable features that can be applied to a broad variety of contexts. Given the vast work that lies ahead, we believe that it is vital to document innovations, actions, and paths undertaken to overcome obstacles to providing quality cancer care.

## CONTEXTUALIZATION OF CANCER CONTROL SYSTEMS

Since the United Nations’ high-level meeting on noncommunicable diseases (NCDs) in 2011, important efforts have been made to provide guidance to policymakers. The adoption of a new cancer resolution at the 70th World Health Assembly in 2017 committed the WHO and Member States to the development, strengthening, implementation, and monitoring of cost-effective national cancer control plans.^14^ A simultaneous update to Appendix 3 of the WHO Global Action Plan^9^ for the Prevention and Control of NCDs (2013-2020) refined actionable targets for countries and delineated Best Buys in NCD control, with specific attention to cost-effectiveness.^15^ The 2015 revision of the WHO Model List of Essential Medicines increased the number of cancer medicines from 30 to 46.^16^ New research also has produced priority advisement to reduce disparities in cancer control.^17,18^ These advances, along with additional health-related gains from the international to the local levels, have laid the foundation for evidence-based policymaking and program development in cancer care and delivery.

## GUATEMALA: BUILDING AND SUSTAINING A PEDIATRIC ONCOLOGY PROGRAM

### Country Background

Guatemala is a mountainous nation in Central America that is home to more than 16 million people, half of whom live in rural areas.^19^ The distribution of income is markedly unequal; 59% of the population lives below the national poverty line^20^ (Table 1). Guatemala’s Mayan indigenous groups comprise 40% to 60% of the population^21,22^ and face extreme socioeconomic barriers to health care.^23^

Guatemala lags behind other Central American countries on government expenditure toward health care^24^ and has the highest private expenditure on health care per capita (83% is out-of-pocket spending). Only 27% of the population is covered by public health insurance.^25^ Signs of poverty are mirrored in high rates of maternal and infant mortality.^26,27^ Half of children in Guatemala younger than age 5 years are chronically malnourished.^28^ The intersection of malnutrition and malignancy as it pertains to survival outcomes and quality of life is vital.^29,30^

### Integration of Cancer Care Into the Health System

In the 1990s, a group of clinicians (including authors of this article) conducted a retrospective study on pediatric cancer outcomes between 1990 and 1995 at the two existing public tertiary care hospitals in Guatemala City: the Roosevelt Hospital and the San Juan de Dios Hospital. The findings were striking: only 100 patients were diagnosed with cancer each year, of 600 expected.^31^ Also, adult oncologists were providing pediatric services, and the 2-year event-free survival rate was only 28% for many diseases now considered curable.^31^ Existing facilities lacked diagnostic tools, systemic therapies, radiotherapy, interventions to treat sepsis, sufficient blood bank support, and appropriate nursing staff. With this needs assessment in hand, the clinicians created a targeted action plan for pediatric cancer care and invited the International Outreach Program (IOP) at St Jude Children’s Research Hospital (SJCRH) to visit Guatemala City to collaborate on this project. Key stakeholders from the Ministry of Health (MOH) and the community participated as well.

Two years later, this group formed the Fundación Ayúdame a Vivir (AYUVI), which led to the following achievements: (1) passing a decree in 1998 to create the Unidad Nacional de Oncología Pediátrica (UNOP; National Pediatric Cancer Unit) as the single center to provide cancer treatment to Guatemalan children; (2) an agreement between UNOP and SJCRH to establish a clinician twinning program to pair American and Guatemalan practitioners to advance care, education, and research^32,33^; and (3) allowing AYUVI to serve as a foundation for advocacy and fundraising. UNOP opened in April 2000 as Gutatemala’s first dedicated pediatric cancer hospital.

The annual budget of UNOP is supported by the AYUVI Foundation (64%) and the MOH (36%); AYUVI covers the infrastructure budget. UNOP reports to the MOH, but it remains a stand-alone facility self-operated and financed without external earmarking. Today, the SJCRH partnership provides 2% of the UNOP operating budget as well as substantial technical support for clinical care.^34^

The number of cases at UNOP has more than quadrupled since its opening (524 patients younger than 18 years old in 2017). Two thousand pediatric patients are on active protocols. In addition, the treatment abandonment rate has been dramatically reduced (41% before UNOP^31^ to 27% in 2001^35^ to now less than 1%), which resulted in a more than doubling of the overall survival rate^36^ (Table 3).

**Table 3:**
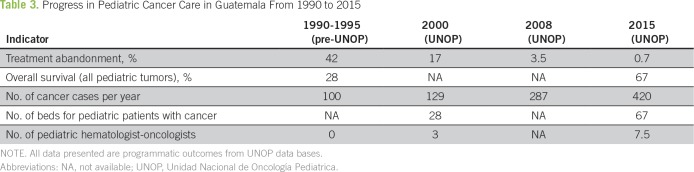
Progress in Pediatric Cancer Care in Guatemala From 1990 to 2015

UNOP provides diagnoses (imaging, pathology), treatments (surgery, chemotherapy, and radiation therapy), supportive care (infection, blood products, nutrition), and psychosocial support (housing, social services, transportation) free of charge.^32^ However, advanced services, such as molecular biology diagnostics, remain beyond reach for the center. There are 70 beds in UNOP (including nine intensive care unit beds). Eight pediatric hematologists-oncologists, as well as several pediatric infectious disease specialists, surgeons, gastroenterologists, and an endocrinologist, are on staff. Continuing medical education for all providers is standard throughout UNOP. A clinical laboratory with microbiology and flow cytometry is open 24/7. Services such as child life, psychology, and palliative care are integrated into patient care from diagnosis. Finally, a pediatric early-warning score program was incorporated to decrease inpatient morbidity and mortality,^37^ and a hospital-based cancer registry has been incorporated into the MOH registry.

UNOP refers patients to external (but integrated) facilities for blood bank, radiotherapy, specific molecular studies, and imaging. Few delays have been recorded because of the strong relationship UNOP maintains with the external facilities. A purchasing manager organizes procurement of medicines from vendors, which has resulted in a stable supply chain. Guatemala is a signatory of the Pan American Health Organization Strategic Fund, which enables the public sector to purchase certain antineoplastic medicines on the WHO Model List of Essential Medicines, as well as supplies and consumables, at discounted rates.^38^ Six data managers are responsible for recording and uploading outcomes data into a global pediatric oncology data base, which ultimately feeds back into UNOP strategic plans as well as to donors and the MOH.^39^

UNOP is also an academic center where medical students from three Guatemalan universities and pediatric residents rotate. In 2003, UNOP and the Guatemalan School of Medicine of Francisco Marroquin University launched a regional fellowship training program with funding from SJCRH, with rotations in the United States, Italy, and Guatemala. This accredited 3-year fellowship program has graduated 21 pediatric hematologist-oncologists from Central and South America. All have returned to their home countries to work in pediatric oncology units.

In Guatemala, the government has begun to implement a National Strategic Plan for Cancer and, in 2015, formed a National Commission of Chronic Nontransmissible Diseases. Separately, pediatric hematologist-oncologists in Guatemala and counterparts from six other Latin American countries launched the Asociación de Hemato-Oncología Pediátrica de Centro América, an organization that annually reviews and agrees upon treatment protocols for nearly a dozen pediatric malignancies.^40^

In Guatemala, many challenges remain, including persistent late-stage diagnosis, suboptimal public sector investment, and limited health care personnel. To maximize efficacy and agility, UNOP solicits annual evaluations through the Asociación de Hemato-Oncología Pediátrica de Centro América, conducts a biweekly AYUVI-UNOP board meeting, and is finalizing its 5-year strategic plan.

## RWANDA: PARTNERING FOR CANCER CONTROL

### Country Background

Rwanda is a landlocked East African country that is home to approximately 11.6 million people, 71% of whom live in rural areas.^41^ The 1994 genocide against the Tutsi dismantled the nation, which resulted in one million lives lost, an entire generation of health care professionals gone or displaced, and a surge of infectious diseases as a result of the broken health care system.^42,43^ When they brought an end to the genocide in 1994, the Rwandan Patriotic Front installed a new, progressive government and declared health care a human right in its Constitution.^44,45^

Although half of Rwandans live on less than $2.00 per day, the gross domestic product per capita has increased significantly from $126 in 1994 to $697 in 2015.^41^ Such progress is a sign of the country’s rapid development toward Rwanda’s Vision 2020 goals.^45^ Life expectancy increased from 28 years in 1995 to 66 years in 2015. Premature mortality in Rwanda decreased so precipitously between 2000 and 2010 that we were unable to find a similar decline in history.^46^ Vaccination coverage went from less than 25% to more than 90%, and maternal mortality and mortality in children younger than age 5 years more than halved. These outcomes have been attributed to accessible national programs, the Rwandan community health worker network (45,000 strong), the enrollment of more than 90% of the population in public health insurance, and the opening of more than 500 health centers.^47^ Yet, despite the exponential increase in the number of clinicians, only three oncologists (one clinical, one radiation, and one pediatric), two hematologists, and one oncoplastic surgeon currently practice in the country.^43,48^

### Integration of Cancer Care Into the Health System

Case documentation efforts in Rwanda in the 1960s to 1980s showed a predominance of cancers with infectious etiologies, but studies were observational, because treatment was not yet available.^49-54^ The burden of cancer in Rwanda has increased since then, in part because of longer life expectancy, greater awareness, and now expanded care and treatment options. A total of 5,812 cancer cases were documented between 2007 and 2014 at Rwandan referral hospitals. In 2010, after major gains in rebuilding its health system, Rwanda welcomed two critical partnerships to address the growing cancer burden.^46,48,55-57^ The first was a 3-year collaboration with Merck to roll out Gardasil, its quadrivalent human papillomavirus vaccine. In 2011, Rwanda rolled out the national program, achieving more than 93% coverage for all doses of the vaccine that first year, and each year since. In 2014 the country transitioned to a partnership with Gavi, the Vaccine Alliance, to maintain this coverage rate.^58^

The second critical partnership led to the 2012 inauguration of the nation’s first cancer center by former US President Bill Clinton. As the country’s only dedicated cancer treatment facility, the Butaro Cancer Center of Excellence (BCCOE) is located in Rwanda’s Northern Province within the Butaro District Hospital. It is a government hospital that is operated with support from Partners in Health (PIH), the Dana-Farber Cancer Institute (DFCI), the Jeff Gordon Children’s Foundation, and additional partners.^55^ Fixed input costs were supported by donations and grants, the land was donated by the government of Rwanda, and operating costs^59^ currently are shared by the MOH, PIH, and DFCI. PIH and DFCI provide additional support in the form of research capacity building, clinical expertise, and training.^56^

The decision to open BCCOE in a rural area was rooted in an existing and strong academic and clinical partnership. The MOH was simultaneously planning to launch four additional cancer treatment units in district hospitals nationwide. The majority of patients at BCCOE initially seek care at their local health center and are referred to BCCOE from a district or referral hospital. A cancer pathology collaboration was introduced at BCCOE in 2012 and included training, anatomic telepathology, installment of new equipment, and expert volunteers.^60^ Samples are processed and analyzed at BCCOE by Rwandan laboratory technicians and two pathologists, with support from DFCI colleagues.^61-63^

In 2015, the MOH worked with DFCI to expand the national drug formulary alongside the WHO Essential Medicines List expansion, which increased the number of antineoplastic agents on its national formulary to 31.^64^ The MOH and DFCI also prepared and approved standardized cancer treatment protocols (15 adult protocols and seven pediatric protocols). BCCOE offers outpatient and inpatient services, diagnosis, surgery, systemic treatment, palliation, and survivorship care, and it provides nutritional and transportation support for patients. Surgeries are performed in Butaro or other hospitals, and the majority of chemotherapy is administered in Butaro. To date, more than 4,000 children and adults have received care at BCCOE regardless of ability to pay.^55^

A vital investment has been the development and refinement of an electronic medical records system to track patient outcomes, which allows for real-time monitoring, quality control, and prospective research.^65^ A number of scientific articles have been published as a result, which document the patient outcomes at BCCOE—implementation science research is a priority of the MOH/DFCI/PIH partnership.^66,67^ Although there are still no oncologists based at BCCOE, their partnerships have allowed for capacity-building and task-shifting.^56,68^ The national Human Resources for Health Program, which jointly launched with the Clinton Health Access Initiative in 2012, also helped advance oncology training.^48^ In addition, several Rwandan physicians currently are enrolled in oncology fellowship programs abroad (eg, Tanzania, Egypt) with contracts to return to Rwanda. In addition, 46 nurses have graduated from a 3-week nursing oncology program, and two have graduated from a 12-week program.^69^

Three major hospitals have cancer pathology, imaging, surgical oncology, and palliative care programs. The main private hospital in the country, King Faisal Hospital, has additional services (ie, computed tomography, magnetic resonance imaging, and chemotherapy). Twenty district hospitals (of 42 total in Rwanda) and four university teaching hospitals are equipped for cervical cancer screening and same-day treatment of precancerous lesions with cryotherapy or loop electrosurgical excision procedure.

Rwanda still faces significant challenges—most notably, the financing of expanded services, such as radiation therapy (RT) and bone marrow transplantation. The Rwanda Military Hospital is opening the country’s first RT facility in 2018. One stopgap solution has been to send patients abroad to receive RT while the MOH raises funds for an RT center. These patients are selected by a multidisciplinary review board on the basis of curative potential, but this selection is an extremely difficult clinical process. An additional challenge is the lack of a cancer registry, although one is in development. Overall, Rwanda has made important strides in the realm of cancer care and recently hosted the 11th International Conference on Cancer in Africa, of AORTIC (African Organization for Research and Training in Cancer).

## VIETNAM: COORDINATING AND ADVANCING THE PUBLIC SECTOR CANCER RESPONSE

### Country Background

Vietnam is a small coastal nation in Southeast Asia with a population of 92.5 million, 67% of whom live in rural areas. The two major cities, Ha Noi and Ho Chi Minh City, are separated by more than 1,000 kilometers and serve as regional health care hubs. Vietnam endured a protracted and troubled history of occupation, colonization, and war. Similar to Rwanda, Vietnam’s economic growth has been remarkable in the last 30 years. Although still considered a lower- to middle-income country, data demonstrate strong annual gross domestic product growth of 5% to 6% for the past two decades along with a steadily increasing life expectancy at birth, which reached 76 years in 2016.

The country’s health care system provides four levels of care: national, provincial, district, and community facilities. This design allows for certain medical needs to be fast-tracked to the national level (eg, cancer care). The main public and private hospitals that are situated in Ha Noi and Ho Chi Minh City are consistently overcrowded; rates of capacity overload range from 150% to 250%. There are two hospital beds per 1,000 people and 1.2 doctors per 1,000 people.^70^ Disparities in access to care vary widely by geography, which contributes greatly to treatment abandonment.

### Cancer Care in Vietnam

According to The World Bank, approximately half of adult men and approximately 1% of adult women in Vietnam smoke tobacco.^70^ It is estimated that 40,000 to 50,000 deaths every year are attributable to this use, and the two leading cancers diagnosed among men in Vietnam are lung and liver cancer. Importantly, tobacco use declined to a low of 47.4% in 2010 because of marketing bans, tax increases, prohibition of smoking in public places, and health education campaigns.

Founded in 1969, the Vietnam National Cancer Hospital in Ha Noi remains the country’s leading cancer facility today. In addition, there are 63 provincial hospitals in the country, 43 of which have oncology departments and trained oncologists to provide cancer care. Across these facilities, a total of 5,000 hospital beds (only 150 of which are in private facilities) are dedicated to patients with cancer; this number reflects prolonged hospital stays for patients who undergo chemotherapy and/or RT, because most Vietnamese patients live too far from the hospitals to receive outpatient therapy. However, cancer care is limited to chemotherapy services at these provincial level hospitals, because targeted therapies are available only at the National Cancer Hospital in Hanoi and are unaffordable to most Vietnamese.

In 2008, the Government approved a National Cancer Control Plan (NCCP) and integrated this effort into the National Program for Health Care by adding training courses for clinicians at the community, district, and provincial levels on cancer prevention, early detection, and screening. A Steering Committee chaired by the Ministry of Health with clinician managers from central and provincial cancer centers was established to implement the NCCP. As part of this plan, general practitioners nationwide received training in basic detection as well as in endoscopy and imaging interpretation for common cancers, including those of the liver, lung, breast, colorectal, cervix, ovarian, and oral cavity. One year later, the Ministry of Health launched a new measure in the plan: a National Cancer Control Network Development initiative, which aims to establish new cancer centers and broaden services within existing facilities. Most recently, in 2015, the government approved its 2015 to 2025 National Strategy for NCD Prevention and Control.

Four national conferences on cancer control are held annually, during which stakeholders discuss progress toward the NCCP and target setting. Priorities at the national level are threefold: (1) enhance mass media and education programs about cancer to raise awareness among the population; (2) improve cancer registries by strengthening data on stage at diagnosis and mortality rates; and (3) expand palliative care programs to the district and provincial levels. An additional priority is hepatitis B vaccination among newborns nationwide, in response to the high liver cancer rates in Vietnam.

Vietnam launched several massive health education efforts, including television campaigns on recognition of early signs and symptoms. Screening for breast and cervical cancer have been rolled out nationwide; colorectal and oral cancer screening are being piloted now in certain communities. To streamline clinical practice, the MOH also has ratified cancer treatment protocols and a national drug formulary for antineoplastics. International partners, including the US National Cancer Institute Center for Global Health, have collaborated on certain elements of the nation’s cancer program as well.

Surgical, medical, and radiotherapeutic services are largely available only in urban settings. Approximately 250 radiation therapists (half are radiation oncologists) practice in the country alongside 136 medical physicists; their available equipment include 39 linear accelerators (LINACs), nine Cobalt machines, and six gamma knife machines. Thirteen LINACs are located in Ha Noi; 12 are in Ho Chi Minh City; in accordance with the plans to extend access regionally, 14 are distributed in other provinces.^71^ From 2010 and 2015, the number of LINACs increased from 13 to 39 because of both government funding and private investments; efforts are underway to ensure that the associated skilled workforce is in place. Progress in treatment outcomes is evidenced by recent hospital-based studies. For example, a study showed that the 3-year overall survival of patients treated for stage III breast cancer was 67.2%^72^ and the 3-year overall survival of patients with stage III non–small-cell lung cancer who were treated with chemotherapy and radiotherapy was 30%.^73^ A palliative care initiative was launched in 2000 at the National Cancer Hospital, and it has grown to include two units at the two central hospitals as well as several provincial hospitals. Hospice care has not yet been established. Local nongovernmental organizations provide certain social support services to patients with cancer at larger facilities.

Although a system is in place for cancer treatment in Vietnam, the infrastructure and workforce required for the national burden of disease remain insufficient. Certain diagnostics—such as immunohistochemistry and molecular analysis—are accessible only at a handful of Vietnam’s hospitals, and the availability of pathologists is limited to a few hospitals. These insufficiencies lead to significant treatment delays and render individual cancer care planning extremely difficult. In addition, geographic disparities that result from the country’s mountainous terrain present the need for a program dedicated to rural cancer detection. Minimal funding for cancer control and high costs of targeted and cytotoxic drugs (even when subsidized) are major challenges. Health insurance in Vietnam does not yet cover all fees, especially advanced technologies and targeted therapies.

## DISCUSSION

The three country cases presented in this article demonstrate the feasibility of establishing cancer care programs in resource-constrained countries. Several principles that underpin the successes deserve highlighting. First, energy and drive from local leadership for the initial demonstrable response to an increasing cancer burden facilitate partner and stakeholder engagement. Second, each case describes the role of partnerships (public and private) and how these can be a vital catalyst to launch and sustain treatment cascades. Third, a focus on professional development that harnesses international expertise for training and education of the health workforce has embedded capacity building in these systems. Strengthening the local workforce and retaining staff are critical, especially given the global competition for skilled practitioners.

Last, all countries exemplify a commitment to geographic and financial access to care. These cases demonstrate the importance of giving early consideration to establishing the entry point into health care at the community level and at the same time to strengthen referral networks to specialty services. In Guatemala, access meant financial affordability and provision of comprehensive services in one facility as well as regional partnerships to leverage purchasing power and clinical expertise. In Rwanda, access meant nationwide interventions and policies, international collaborations to advance treatment options, and provision of treatment regardless of ability to pay. In Vietnam, access meant improvement in geographic equity by strengthening the two main public facilities in the north and south as well as engagement of a broad set of stakeholders in planning efforts. While Guatemala’s case sheds light on progress driven through the public-private interface and nongovernmental actors, Rwanda’s and Vietnam’s examples highlight the progress of government-led health care delivery in nations recovering from immeasurable devastation. Finally, and perhaps most importantly, the policy decision that was engrained in each program from the start was the restoration of dignity in the care of patients with cancer. This is no small decision, although it is often overlooked. All three countries demonstrate that excellent cancer care can and should be provided to all, including those who are impoverished or marginalized, without acceptance of a double standard. It is our hope that this article provides a framework of evidence and the programmatic elements to other countries looking to create or grow their own initiatives.

Emerging robust models of care can inspire both national cancer planning and peers in other countries who may encounter related challenges. This only can happen, though, if such efforts are documented and publicized. Implementation research, research capacity building, and dissemination of findings will be critical to shape national cancer control planning over time. We call on governments and program planners to share their experiences to maximize knowledge sharing and to allow for informed progression toward the 2025 global goals. We are convinced that there is a future in which where you are diagnosed with cancer does not determine whether you survive, and we hope that this article will provide insights to countries who share this belief.

**Panel**

**Key Features of Success, Organized by the WHO “Six Building Blocks for Health Systems”**^13^

This panel outlines the core elements that have enabled Guatemala, Rwanda, and Vietnam to establish robust cancer treatment facilities. The following features are drawn from examples from all three countries.

**WHO Building Block 1: Service Delivery**

- Simultaneous centralization and decentralization: Centralization of comprehensive services to regionally distributed facilities; decentralization of basic services and diagnostic capacity for referrals
- Focusing efforts on excellence of care (not accepting a double standard in delivery nor outcomes)
- Building strong partnerships with academic medical centers in high-income countries (HICs)
- Remote consults from oncologists and pathologists in HICs via tumor board or online systems
- Respect for the cultural characteristics of the local population in developing programs

**WHO Building Block 2: Health Workforce**

- Retaining cancer specialists in country through policies or incentives
- Welcoming volunteer experts to support program development according to national agenda
- Creating regional training centers for oncologists, attracting talent and retaining skilled personnel
- Offering continuing medical education at all levels of healthcare providers in cancer care
- Developing training programs for non-MDs (eg, nursing oncology, cancer epidemiology)
- Building and maintaining twinning programs with high-income countries for capacity transfer

**WHO Building Block 3: Information**

- Baseline national survey at initiation to assess needs and gaps in cancer care
- Use of WHO’s CANREG-5 to create national or hospital-based registry
- Establishment of electronic, prospective records to monitor and document outcomes
- Integrating implementation science into policy, strategic planning, and program development
- Strengthening academic collaborations with centers in high income countries to advance education and build a joint research agenda that enables professional development on both sides

**WHO Building Block 4: Medical Products, Vaccines, Technologies**

- Collaboration with regional neighbors to leverage purchasing power for medicines
- Adding essential antineoplastics to the national formulary for availability in the public sphere
- Rollout of the human papillomavirus vaccine nationwide; rollout of tobacco prevention program nationwide
- Private sector negotiations to increase availability of medicines, diagnostics, and radiotherapy

**WHO Building Block 5: Financing**

- Ensuring financial sustainability through continuous increasing of government contribution
- Establishing a separate nonprofit model or partner to enable corporate fundraising
- Creativity and agility in sourcing for financing partners to advance cancer treatment agenda

**WHO Building Block 6: Leadership/Governance**

- Adoption and implementation of a legal framework for noncommunicable diseases including cancer
- Regular interval symposia across stakeholders to discuss key policies and programs
- Mass communication campaigns, especially regarding preventable cancers
- Central orchestration of cancer control policy by the Ministry of Health
- Institutionalized collaboration between policy-makers and clinician leaders to ensure alignment
- Providing care at limited or no cost to patients including support for wraparound services
- Rapid and nimble implementation of national strategies for noncommunicable disease prevention and control
